# Supplementary material for: Reduced neural activity but improved coding in rodent higher-order visual cortex during locomotion
Source: Nat Commun. 2022 Mar 30;13:1676. doi: 10.1038/s41467-022-29200-z (PMC8967903; doi:10.1038/s41467-022-29200-z)
Supplement: Supplementary file 1 — Supplementary Information [file 41467_2022_29200_MOESM1_ESM.pdf]

Supplementary Information

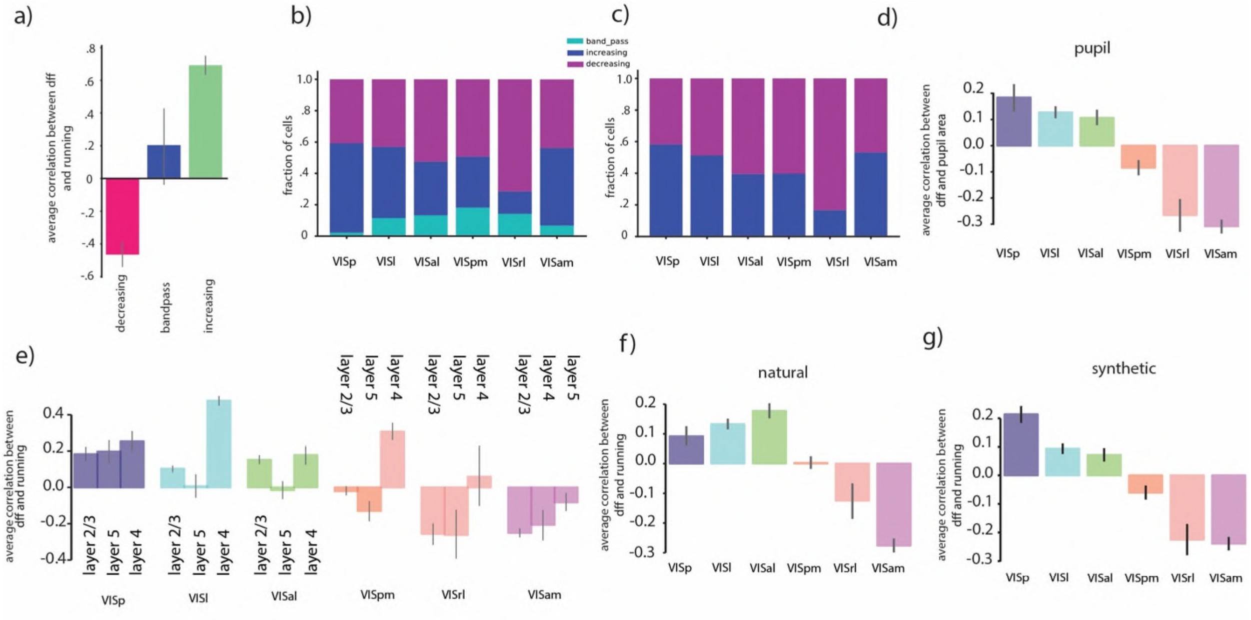

h)

a)

	layer 2/3	layer 4	layer 5	
VISp	802, 326, 103, 143	257, 35, 10, 12	175, 59, 9, 17	natural
	1004, 306, 95, 150	479, 105, 13, 65	181, 102, 18, 56	artificial
VISl	2708, 1543, 472, 684	656, 212, 27, 108	111, 68, 22, 21	natural
	2622, 1559, 538, 624	540, 355, 41, 208	181, 102, 18, 56	artificial
VISal	1346, 531, 125, 273	201, 146, 34, 79	329, 151, 52, 60	natural
	1346, 772, 238, 350	201, 90, 34, 40	329, 239, 102, 90	artificial
VISpm	1817, 847, 317, 275	272, 208, 49, 125	329, 151, 52, 60	natural
	1905, 628, 271, 181	183, 165, 37, 97	182, 164, 79, 41	artificial
VISrl	112, 92, 49, 20	55, 26, 5, 17	14, 12, 4, 1	natural
	112, 95, 42, 15	201, 90 34, 40	14, 13, 4, 2	artificial
VISam	966, 770, 454, 172	149, 95, 31, 23	60, 48, 29, 9	natural
	992, 846, 458, 204	60, 57, 32, 16	95, 69, 23, 16	artificial
	all, tuned, neg, pos	all, tuned, neg, pos	all, tuned, neg, pos	

i)

b)

	layer 2/3	layer 4	layer 5	
VISp	1004, 306, 90, 151	479, 97 14, 52	181, 98, 23, 49	stimulus
	841, 301, 140, 101	464, 38, 3, 17	168, 48, 4, 31	spontaneous
VISl	2682, 1697, 519, 752	683, 355, 27, 246	111, 105, 38, 40	stimulus
	2131, 892, 380, 308	573, 224 26, 137	168, 48, 4, 100	spontaneous
VISal	1380, 790, 219, 376	201, 150, 37, 70	329, 239, 92, 90	stimulus
	1112, 516, 251, 112	247, 147, 74, 42	289, 165, 82, 51	spontaneous
VISrl	112, 103, 53, 18	27, 10, 2, 3	14, 14, 7, 2	stimulus
	259, 120, 30, 54	96, 36, 10, 7	14, 11, 6, 1	spontaneous
VISpm	1905, 810, 299, 280	183, 169, 37, 99	182, 158, 69, 44	stimulus
	1379, 526, 243, 162	272, 204, 50, 101	159, 105, 54, 30	spontaneous
VISam	1014, 881, 495, 222	149, 104, 32, 26	60, 57, 31, 14	stimulus
	726, 477, 277, 79	58, 34, 20, 8	60, 40, 22, 7	spontaneous
	all, tuned, neg, pos	all, tuned, neg, pos	all, tuned, neg, pos	

Supplemental Figure 1 a. Average correlation coefficient for neurons monotonically tuned to running determined to either be band-pass, positively tuned to running, or negatively tuned to running based on fitting of a constrained Gaussian function (see methods) **b.** Fraction of neurons displaying different tuning types to running, with tuning type calculated via model fitting as in a. **c)** Ratio of neurons displaying increasing and decreasing tuning to running, calculated via model fitting as above. **d**. Correlation coefficient between pupil diameter and calcium activity in monotonically correlated neurons. **e**. Average correlation coefficient with running for monotonically tuned neuron, individual cre-lines displayed, calculated across all stimuli types. **f**. Pearson's correlation coefficient between running speed and dF/F in each region, calculated only natural stimuli (natural scenes, natural movies) were displayed. **g**. Same as e. but calculated only when synthetic stimuli (e.g. gratings, noise) were displayed. **h**. Number of tuned neurons displaying monotonic increasing vs. monotonic decreasing tuning to running, split out by natural and artificial stimulus types. **i.** Number of neurons tuned to running, split out by periods with and without stimulus. All cell numbers for preceding panels are found in h and i. All error bars are 95 confidence intervals on the mean estimate, determined by 1000 bootstraps.

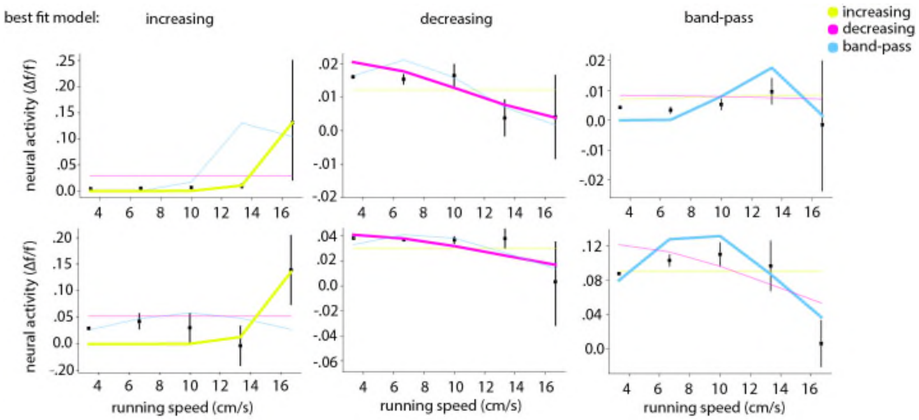

Supplemental Figure 2. Examples of cells whose activity are best fit by increasing, decreasing, and band-pass gaussian models, respectively. In each plot the average neural activity (in black), and all fit models (yellow, pink, and blue) are shown. Plotted neural data are from a held-out test set, model fits are the MLE model from the training set. Best fit model was determined by lowest residual on the test set, across 10 cross-validation folds. All cells are from layer 2/3 of primary visual cortex. Models were fit on data collected while any of the visual stimuli were presented. **Error bars are standard error margin for observed running speed data.**

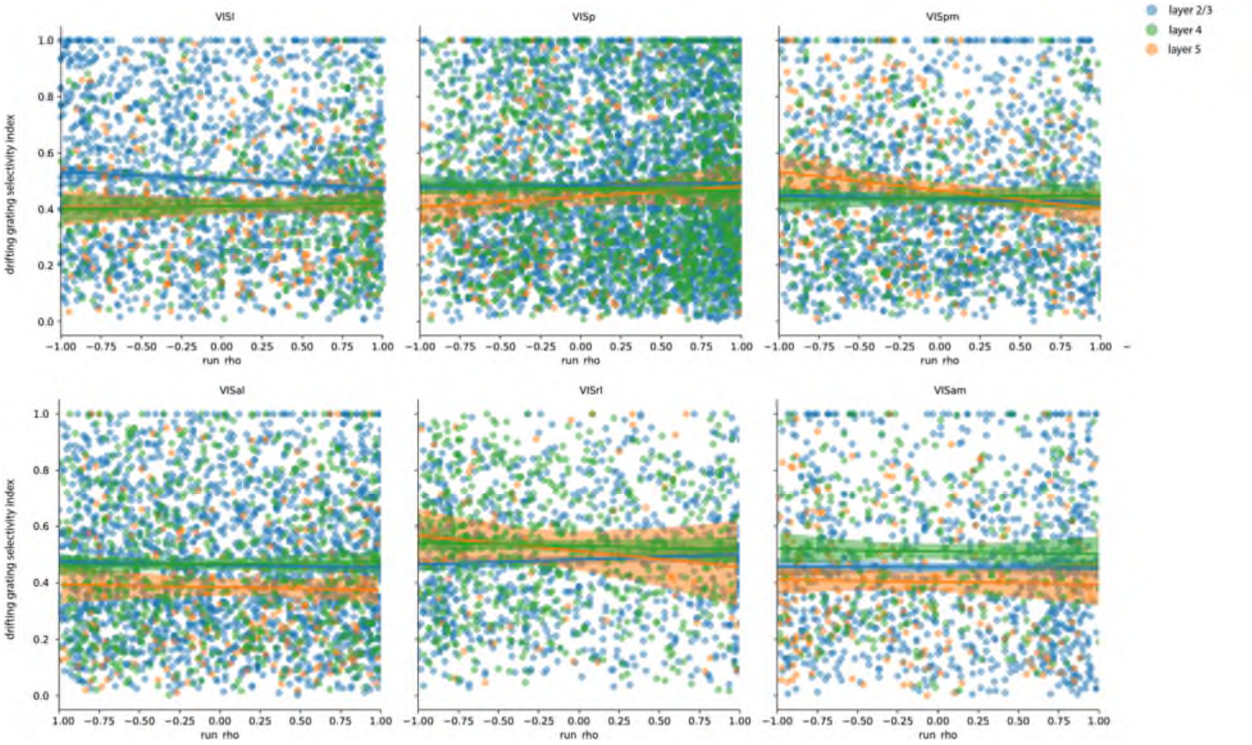

Supplemental Figure 3. *Correlation between drifting grating selectivity and correlation to running, in all neurons, separated by recording layer. Cell numbers: VISp l23: 2440, l4: 515, l5: 1544, VISl l23: 7052, l4: 277, l5: 2173, VISpm l23 4539, l4 :702, l5: 579. VISal: l2/3 4037, l4 746, l5 643, VISam l23 3027, l4 201, l5 732, VISrl l23 737, l4 41, l5 460*

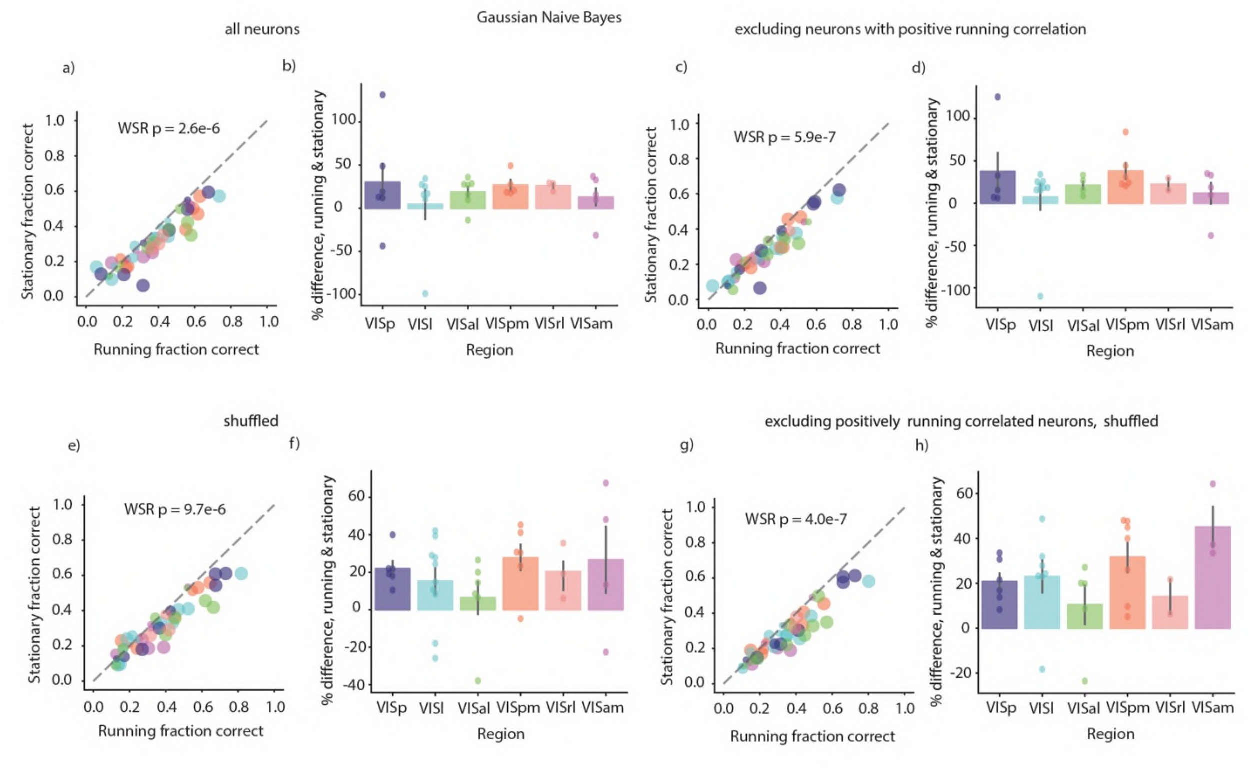

Supplemental Figure 4 Decoder: Gaussian Naïve Bayes. Decoding performance during running and stationary periods. **a**. Average fraction of correctly classified visual stimuli during running and stationary periods (average over ten 50:50 train/test splits). Each data point is an individual experiment. Colors indicate brain region recorded. **b**. Data from **a** displayed separated into visual regions in dataset, only including experiments in which the difference between running and stationary periods was significant (in either direction). Each dot is an individual experiment. **c,d**. Same as **a, b**. but excluding neurons that increase their activity during running. **e, f**. Same as a. but trial-shuffled to remove noise correlations. **g, h.** Same as a. but excluding neurons that increase their activity during running and trial-shuffled to remove noise correlations. All statistics Wilcoxon signed rank test. Cell numbers and mouse numbers supplemental table 1. Error bars are 95% confidence intervals on the mean estimate, 1000 bootstraps.

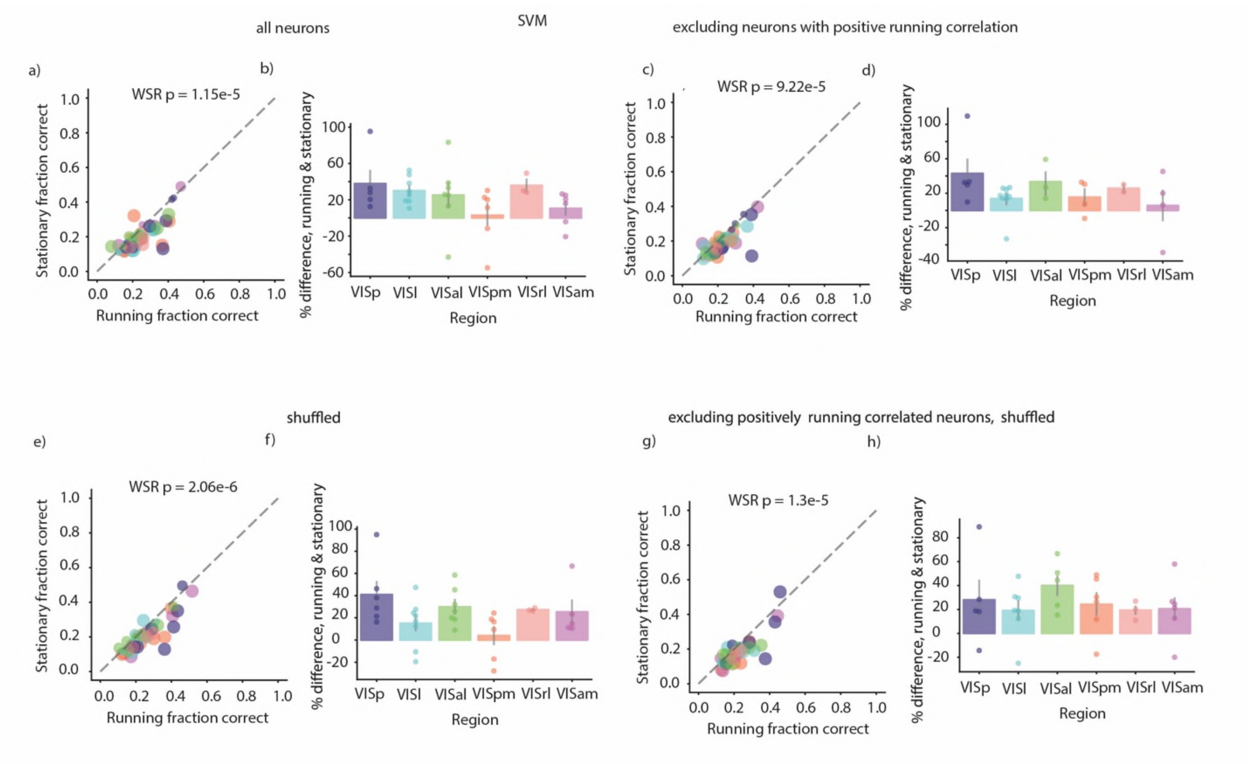

Supplemental Figure 5. Decoding performance (SVM Support Vector Machinne) during running and stationary periods. **a**. Average fraction of correctly classified visual stimuli during running and stationary periods (average over ten 50:50 train/test splits). Each data point is an individual experiment. Colors indicate brain region recorded. **b**. Data from **a** displayed separated into visual regions in dataset, only including experiments in which the difference between running and stationary periods was significant (in either direction). Each dot is an individual experiment. **c,d**. Same as **a, b**. but excluding neurons that increase their activity during running. **e, f**. Same as a. but trial-shuffled to remove noise correlations. **g, h.** Same as a. but excluding neurons that increase their activity during running and trial-shuffled to remove noise correlations. All statistics Wilcoxon signed rank test. Cell numbers and mouse numbers supplemental table 1. Error bars are 95% confidence intervals on the mean estimate, 1000 bootstraps.

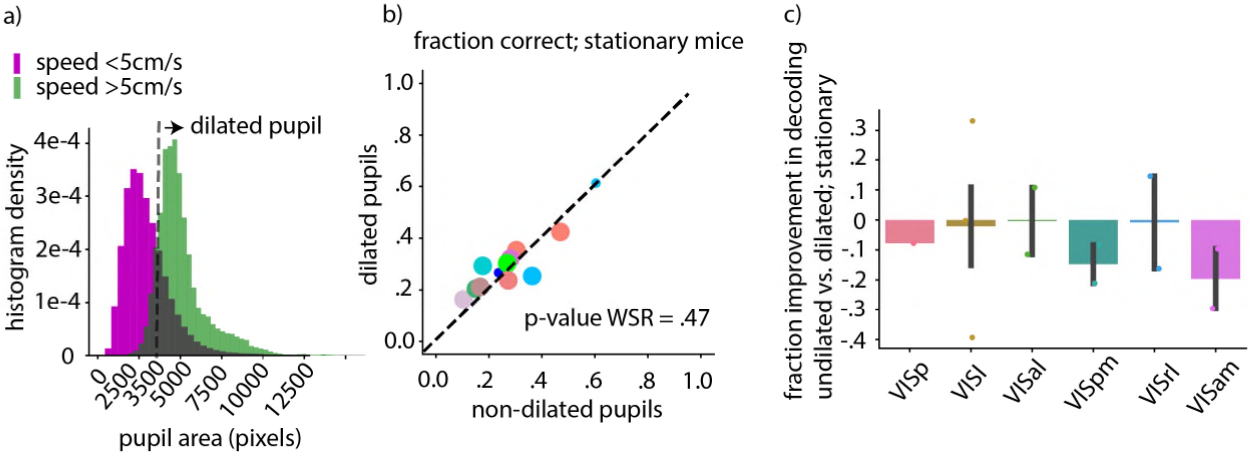

Supplemental Figure 6. a) distribution of pupil diameter during running and stationary periods. b) fraction of correctly classified trials comparing periods where mice were stationary and had dilated pupils (defined as pupil area greater than 3500 pixels, the pupil area which separates the running and stationary pupil distributions) and those stationary trials where mice had undilated

pupils. C. Same as b. except broken out by region. In **b.** size of circle represents statistical effect size. Cell numbers and mouse numbers supplemental table 1. Error bars are 95% confidence intervals on the mean estimate, 1000 bootstraps.

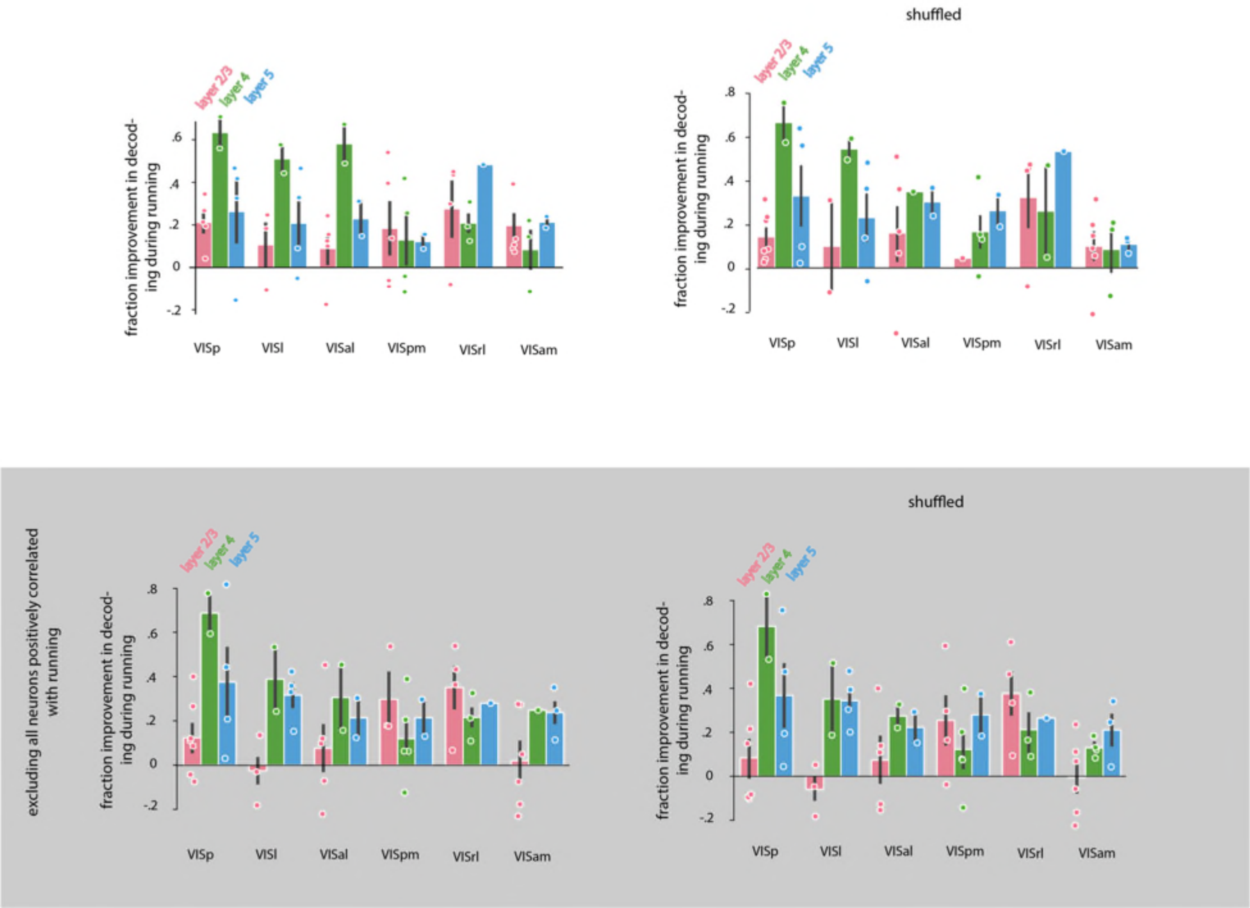

Supplemental Figure 7. Difference between running and stationary decoding (same as Figure 3b, etc.) except broken out by visual region and cre-line. Cell numbers and mouse numbers supplemental table 1. Error bars are 95% confidence intervals on the mean estimate, 1000 bootstraps.

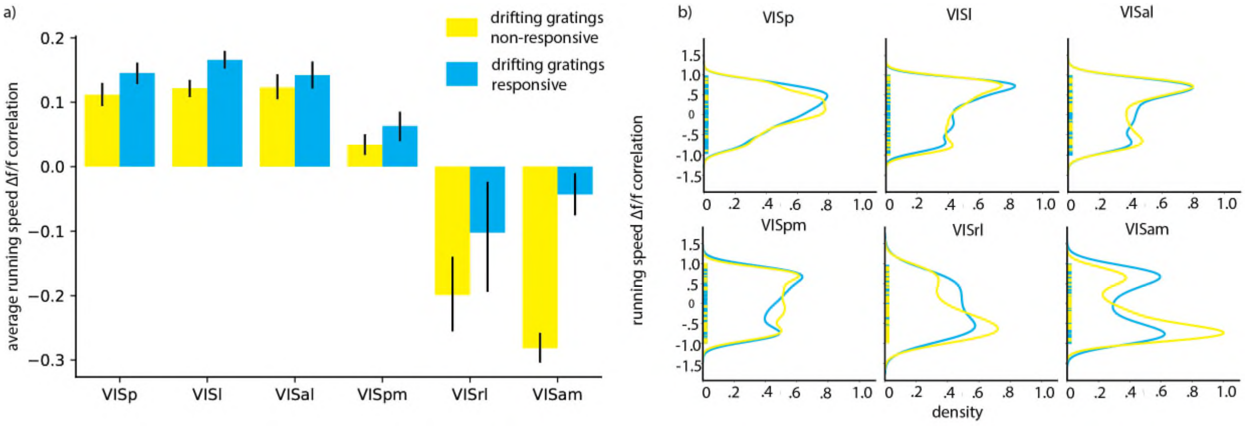

Supplemental Figure 8. A) average correlation coefficient between running and neural activity, grouped by responsiveness to drifting gratings. A neuron is considered responsive to drifting

gratings if it passes an Anova with $p < 0.05$ comparing the distribution of responses to all stimuli, including the blank stimuli, with the null hypothesis that all distributions are the same. (B) the histogram density of running speed – neural activity correlation coefficients, split up by region and responsiveness to the drifting gratings stimuli. Cell numbers: Drifting gratings responsive: VISp 272, VISl 696, VISpm 331, VISrl 39, VISam 390. Drifting gratings non-responsive: VISp 229, VISl 1461, VISpm 806, VIS4l 88, VISam 652. Error bars are 95% confidence intervals on mean estimate determined by 1000 bootstraps.

**VISp**					
**layer 2/3**		**layer 4**		**layer 5**	
**All**	**non-pos**	**All**	**non-pos**	**All**	**non-pos**
353	263	79	65	61	52
292	237	43	36	101	90
215	110	42	31		
266	211	60	51		
200	179	100	90		
240	160				
95	58				
**VISl**					
**layer 2/3**		**layer 4**		**layer 5**	
**All**	**non-pos**	**All**	**non-pos**	**All**	**non-pos**
188	140	191	113	88	66
108	81	200	115	52	33
293	238			28	28
109	60			50	37
253	153				
**VISal**					
**layer 2/3**		**layer 4**		**layer 5**	
**All**	**non-pos**	**All**	**non-pos**	**All**	**non-pos**
76	68	89	80	21	15
159	123	234	206	96	72
207	144				
47	43				
168	158				
**VISpm**					
**layer 2/3**		**layer 4**		**layer 5**	
**All**	**non-pos**	**All**	**non-pos**	**All**	**non-pos**

128	89	79	65	61	52
85	70	43	36	101	90
94	76	42	31		
106	81	60	51		
232	168	100	90		
**VISrl**					
**layer 2/3**		**layer 4**		**layer 5**	
**All**	**non-pos**	**All**	**non-pos**	**All**	**non-pos**
82	74	261	234	9	8
34	24	217	207	13	10
112	100	146	113		
125	102				
**VISam**					
**layer 2/3**		**layer 4**		**layer 5**	
**All**	**non-pos**	**All**	**non-pos**	**All**	**non-pos**
79	66	32	26	75	69
181	129	93	81	181	129
239	160	50	38	239	160
112	87	58	52	112	87
82	67			82	67
35	27			35	27
118	79			118	79

Supplemental Table 1: Cell numbers included in decoding analysis. Each line in each layer is a separately recording experiment, non-pos refers to cell numbers after excluding neurons positively correlated neurons. Thus each entry corresponds to one data point in all decoding analyses.

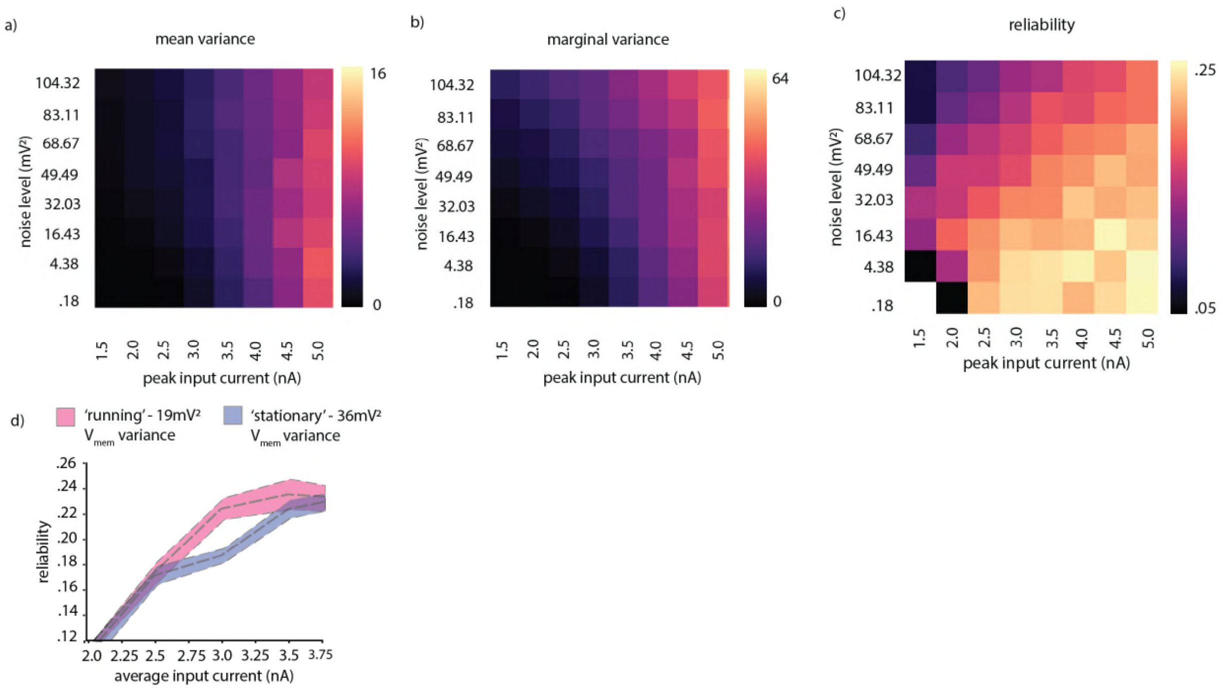

Supplemental Figure 9 a. Variance of class means versus noise level and peak input current in LIF simulations. **b**. Marginal variance versus peak input current and noise level in LIF simulations. **c**. Reliability vs noise level and peak input current. **d.** Reliability vs. average input current during running and stationary noise levels, with resting membrane voltage also adjusted to -68 mV and -70 mV during running and stationary periods, respectively. Each datapoint is averaged over 10 independent re-runs of the model.

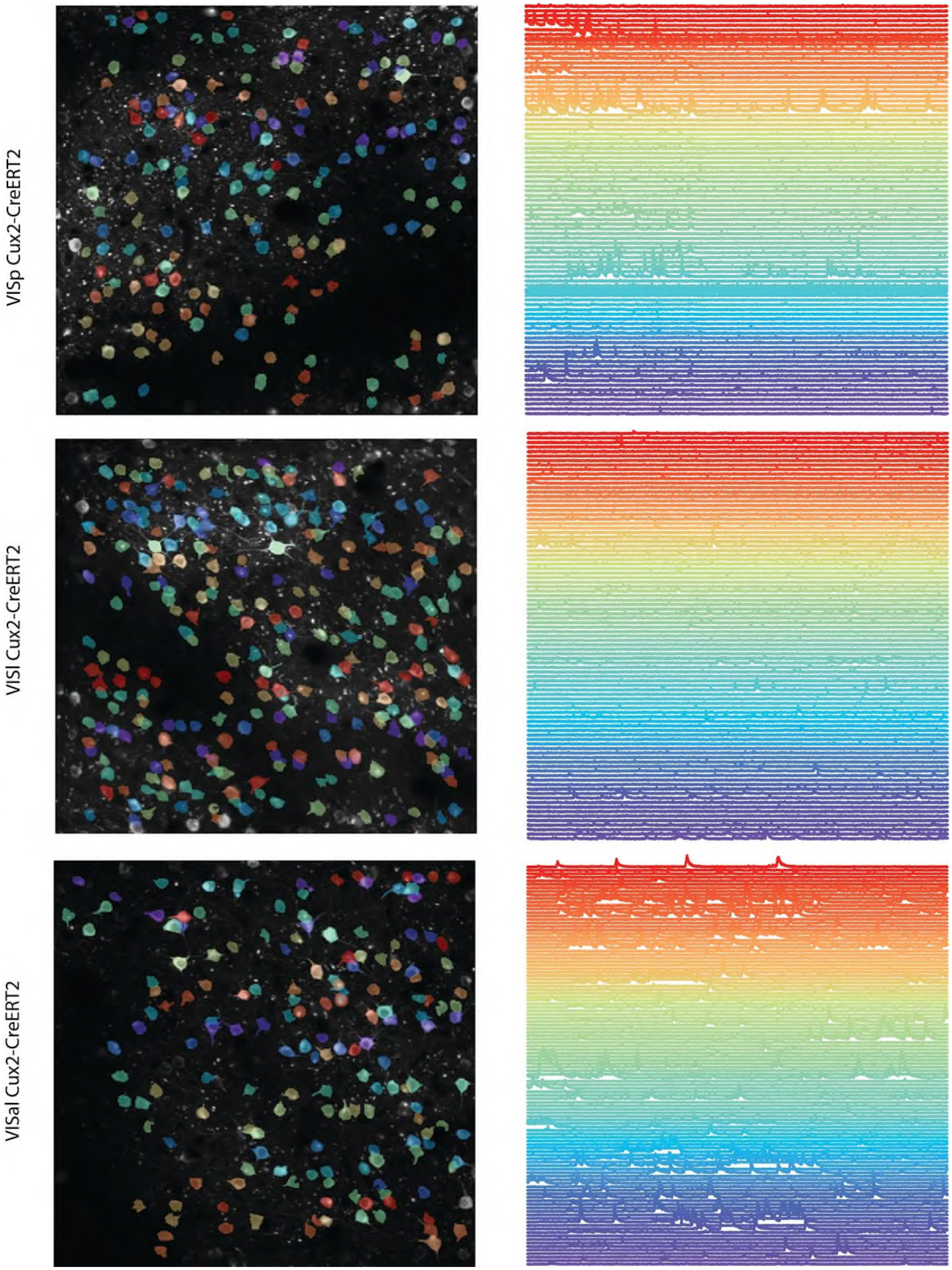
VISp Cux2-CreERT2
VISl Cux2-CreERT2
VISal Cux2-CreERT2

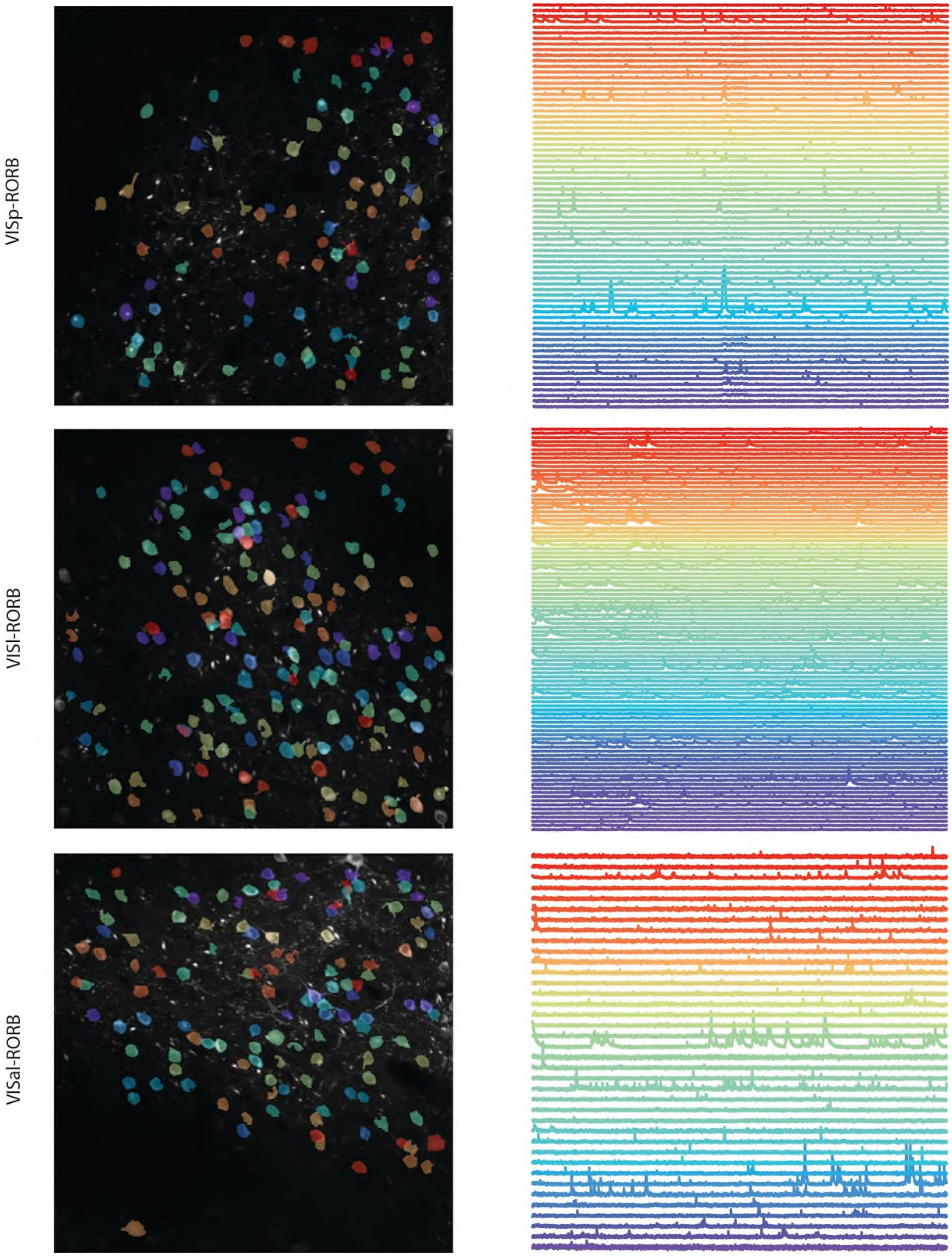

Supplemental Figure 10*.* Example cell traces and ROIs. Colors in cell mask ROI images (left) correspond with colors in cell trace df/f images (right). Each field of view (left) is 512um by 512um.
