# Supplementary material for: Reduced neural activity but improved coding in rodent higher-order visual cortex during locomotion
Source: Nat Commun. 2022 Mar 30;13:1676. doi: 10.1038/s41467-022-29200-z (PMC8967903; doi:10.1038/s41467-022-29200-z)
Supplement: Supplementary file 4 — Reporting Summary [file 41467_2022_29200_MOESM4_ESM.pdf]

nature research

Corresponding author(s): JW Pillow

Last updated by author(s): 1/24/2022

Reporting Summary

Nature Research wishes to improve the reproducibility of the work that we publish. This form provides structure for consistency and transparency in reporting. For further information on Nature Research policies, see our Editorial Policies and the Editorial Policy Checklist.

Statistics

For all statistical analyses, confirm that the following items are present in the figure legend, table legend, main text, or Methods section.

n/a	Confirmed	
☐	☒	The exact sample size (n) for each experimental group/condition, given as a discrete number and unit of measurement
☐	☒	A statement on whether measurements were taken from distinct samples or whether the same sample was measured repeatedly
☐	☒	The statistical test(s) used AND whether they are one- or two-sided<br>*Only common tests should be described solely by name; describe more complex techniques in the Methods section.*
☐	☒	A description of all covariates tested
☐	☒	A description of any assumptions or corrections, such as tests of normality and adjustment for multiple comparisons
☐	☒	A full description of the statistical parameters including central tendency (e.g. means) or other basic estimates (e.g. regression coefficient) AND variation (e.g. standard deviation) or associated estimates of uncertainty (e.g. confidence intervals)
☐	☒	For null hypothesis testing, the test statistic (e.g. F, t, r) with confidence intervals, effect sizes, degrees of freedom and P value noted<br>*Give P values as exact values whenever suitable.*
☐	☒	For Bayesian analysis, information on the choice of priors and Markov chain Monte Carlo settings
☐	☒	For hierarchical and complex designs, identification of the appropriate level for tests and full reporting of outcomes
☐	☒	Estimates of effect sizes (e.g. Cohen's d, Pearson's r), indicating how they were calculated

Our web collection on statistics for biologists contains articles on many of the points above.

Software and code

Policy information about availability of computer code

Data collection	Data were collected by the Allen Institute Brain Observatory.
Data analysis	Data were analyzed using the Brain Observatory SDK, Sci-Kit Learn, and custom written Python code that is available at github.com/achristensen56. Data were analyzed using python 3.7.9, allensdk 2.3.2, matplotlib 3.3.2, pandas 0.25.3, scikit-learn 0.23.2, scipy: 1.5.2, seaborn: 0.11.0

For manuscripts utilizing custom algorithms or software that are central to the research but not yet described in published literature, software must be made available to editors and reviewers. We strongly encourage code deposition in a community repository (e.g. GitHub). See the Nature Research guidelines for submitting code & software for further information.

Data

Policy information about availability of data

All manuscripts must include a data availability statement. This statement should provide the following information, where applicable:

- Accession codes, unique identifiers, or web links for publicly available datasets
- A list of figures that have associated raw data
- A description of any restrictions on data availability

Data is freely available via the Allen Institute Brain Observatory.

Field-specific reporting

Please select the one below that is the best fit for your research. If you are not sure, read the appropriate sections before making your selection.

☒ Life sciences ☐ Behavioural & social sciences ☐ Ecological, evolutionary & environmental sciences

For a reference copy of the document with all sections, see nature.com/documents/nr-reporting-summary-flat.pdf

Life sciences study design

All studies must disclose on these points even when the disclosure is negative.

Sample size

No sample size calculation was done. We used all relevant data from the publicly available dataset we analyzed and effect sizes were estimated via bootstrapping.

Data exclusions

We excluded mice who did not have at least 50 trials of running and 50 trials of stationary data from our analysis. This was to ensure we had adequate sampling of both running and stationary periods across all stimuli presented. The numbers of excluded datasets are below:

VISpm (9 / 21 experiments used)
VISp (8 / 24 experiments used)
VISal(8 / 20 experiments used)
VISl (12 / 23 experiments used)
VISrl (3 / 6 experiments used)
VISam (7 /15 experiment used)

Replication

We did not independently replicate this study. The data were collected as a part of a large scale effort from the Allen Institute for Brain Science, and not by us. We do not have the ability to replicate the experimental paradigm.

Randomization

Data were randomly sub-sampled for experimental groups to create even numbers of data points in each group. We did not e.g. randomize subjects into experimental conditions because the experimental conditions were self-generated, to be concrete the mice chose when they run, not us. We balanced data for covariates like which stimuli class was presented by sub-sampling to ensure all classes were present in both running and stationary datasets, to control for the possibility mice chose to ran more during some stimuli than others.

Blinding

No blinding was performed. We did not perform the data collection, or any experiments where subjective results were reported.

Reporting for specific materials, systems and methods

We require information from authors about some types of materials, experimental systems and methods used in many studies. Here, indicate whether each material, system or method listed is relevant to your study. If you are not sure if a list item applies to your research, read the appropriate section before selecting a response.

Materials & experimental systems

n/a	Involved in the study	
☒	☐	Antibodies
☒	☐	Eukaryotic cell lines
☒	☐	Palaeontology and archaeology
☒	☐	Animals and other organisms
☒	☐	Human research participants
☒	☐	Clinical data
☒	☐	Dual use research of concern

Methods

n/a	Involved in the study	
☒	☐	ChIP-seq
☒	☐	Flow cytometry
☒	☐	MRI-based neuroimaging
